# Moderate neuroprotection of combination cell therapy in fetal growth restricted newborns at postnatal day 10

**DOI:** 10.1093/stcltm/szag019

**Published:** 2026-04-20

**Authors:** Kirat K Chand, Kate Beecher, Rachel Nano, Seen-Ling Sim, Jane Sun, Peytn Stokes-Marshall, Lillian Macfarlane, John Luff, Hannah Musco, Paul B Colditz, Kiarash Khosrotehrani, Jatin Patel, Julie A Wixey

**Affiliations:** UQ Centre for Clinical Research, Faculty of Health, Medicine and Behavioural Sciences, The University of Queensland, Brisbane, QLD 4029, Australia; UQ Centre for Clinical Research, Faculty of Health, Medicine and Behavioural Sciences, The University of Queensland, Brisbane, QLD 4029, Australia; Frazer Institute, The University of Queensland, Woolloongabba, QLD 4102, Australia; Frazer Institute, The University of Queensland, Woolloongabba, QLD 4102, Australia; Frazer Institute, The University of Queensland, Woolloongabba, QLD 4102, Australia; UQ Centre for Clinical Research, Faculty of Health, Medicine and Behavioural Sciences, The University of Queensland, Brisbane, QLD 4029, Australia; UQ Centre for Clinical Research, Faculty of Health, Medicine and Behavioural Sciences, The University of Queensland, Brisbane, QLD 4029, Australia; UQ Centre for Clinical Research, Faculty of Health, Medicine and Behavioural Sciences, The University of Queensland, Brisbane, QLD 4029, Australia; UQ Centre for Clinical Research, Faculty of Health, Medicine and Behavioural Sciences, The University of Queensland, Brisbane, QLD 4029, Australia; UQ Centre for Clinical Research, Faculty of Health, Medicine and Behavioural Sciences, The University of Queensland, Brisbane, QLD 4029, Australia; Perinatal Research Centre, Royal Brisbane and Women’s Hospital, Brisbane, QLD 4029, Australia; Frazer Institute, The University of Queensland, Woolloongabba, QLD 4102, Australia; Frazer Institute, The University of Queensland, Woolloongabba, QLD 4102, Australia; Faculty of Health, Queensland University of Technology, School of Biomedical Sciences, Brisbane, QLD 4059, Australia; UQ Centre for Clinical Research, Faculty of Health, Medicine and Behavioural Sciences, The University of Queensland, Brisbane, QLD 4029, Australia

**Keywords:** fetal growth restriction, stem cells, brain injury, neonate

## Abstract

Infants with fetal growth restriction (FGR) are at increased risk of adverse neurodevelopmental conditions, including motor, learning, and behavioral deficits. There are currently no treatments to protect the FGR newborn from lifelong neurological conditions. We have previously reported neuroprotective potential of a single dose of combined mesenchymal stromal cells and endothelial colony-forming cells (ECFCs) therapy, termed cECFC, isolated from healthy human term placenta, in treating brain injury in a preclinical model of FGR. We administered cECFCs to newborn FGR pigs and survived to postnatal day 4 (2-week human equivalence). We reported improved gray and white matter integrity, reduced glial-mediated inflammation and improved microvasculature. Here, we aimed to examine whether this novel therapy presented sustained efficacy in newborn pigs that survived to postnatal day 10 (1-month human equivalence). We determined a single dose of cECFC treatment affords moderate neuroprotective capacity in the cortex but limited efficacy in the periventricular white matter. We also report minimal modulation of the inflammatory environment, with ongoing glial activation observed in most regions examined. Our data suggest a diminution in efficacy of single-dose cECFC 10 days after administration. We propose multiple doses of cECFCs may be required to maintain neuroprotective capacity during early post–natal life in FGR newborns. Overall, these findings demonstrate the importance of extended pre–clinical studies to determine the efficacy of treatments prior to translation to clinical trials.

## Graphical abstract

**Graphical Abstract:**
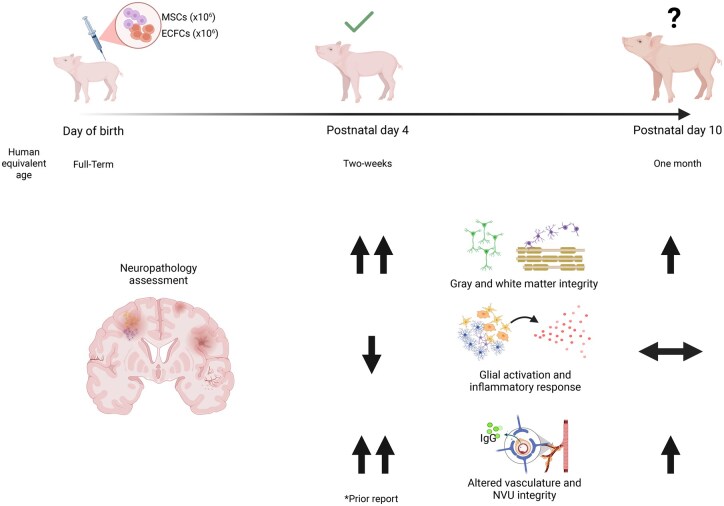

Significance statement We have shown that a single dose of placental stem cells (endothelial colony-forming cells and mesenchymal stromal cells) administered at birth can reduce fetal growth-restricted brain injury. In this study, we investigated whether these positive outcomes persist. We demonstrate moderate neuroprotection and observed a limited effect on inflammation contrasting with the robust response reported in the short-term study. Our findings suggest a loss in therapeutic efficacy by 10 days post–administration. This study not only advances our knowledge and understanding of combined stem cell therapies in treating fetal growth restriction neonates but also highlights the need for ongoing studies to ensure long-term therapeutic efficacy.

## Introduction

Fetal growth restriction (FGR) is a common complication in pregnancy where an unborn baby fails to reach its expected growth potential. FGR is estimated to affect up to thirty million pregnancies per year.^1^ Neurodevelopmental delays are observed in 24%-53% of FGR infants at 2 years of age,^2^ with no therapeutics available to protect the FGR newborn. Studies in preclinical models of FGR demonstrate inflammation may significantly contribute to neuropathology including cellular changes to gray and white matter, altered neurovascular integrity and impaired vascularization.^3-6^ Combined cell therapy is a viable strategy to treat newborn FGR brain injury as these cells may afford neuroprotection through their multi–modal actions such as immune modulation, angiogenesis, and augmentation of neurogenesis. Two well-characterized cell types include mesenchymal stromal cells (MSCs) and endothelial colony-forming cells (ECFCs), which have potent immunomodulatory effects and promote angiogenesis.^7^ An important issue, however, with treatments using ECFCs individually, is the potential for a reduction in engraftment and survival in a host without immunosuppression. Studies have shown co-administration of MSCs and ECFCs may have synergistic benefits with enhancement of cell modulatory capacity leading to better outcomes over monotherapy.^8-10^ MSCs are known for their immunosuppressive action and therefore provide an environment for ECFCs to home to, survive, engraft, and build or restore the damaged vascular network.

Our previous findings provided evidence that postnatal intervention using a combination of placental-derived MSCs and ECFCs, termed cECFC, affords neuroprotection in the FGR newborn pig at postnatal day 4 (2-week human equivalent).^11^ The study demonstrated recovery of key gray and white matter cellular elements, ameliorated glial-associated pro-inflammatory profiles, improved vascularization, and blood–brain barrier (BBB) integrity. The potential of cell therapies in treating newborn brain pathologies has led to numerous acute studies demonstrating efficacy; however, there are limited reports extending these findings beyond the immediate neonatal period.^7^ The present study aimed to extend our positive short-term findings and investigate the therapeutic potential of cECFC administration in the newborn FGR pig by examining brain outcomes at postnatal day 10, the equivalent of approximately 1 month in the human newborn.

## Materials and methods

Newborn Large White FGR (<10th percentile birth weight) and normally grown (NG) piglets (10-90th percentile) (<18 h) were collected from a commercial piggery on the day of birth, monitored and cared for at the Herston Medical Research Centre (HMRC) until the day of euthanasia on postnatal day 10 (P10). Piglets were divided into 4 experimental groups with pigs randomly assigned to treatment groups under double-blinded conditions: NG (*n* = 10), FGR (*n* = 10), FGR + cECFC (*n* = 8), and FGR + MSC (*n* = 8). Litter-matched piglets were obtained from multiple sows with males and females in each group. Key physiological parameters are summarized in Table S1. This study was approved by The University of Queensland Animal Ethics Committee (2022/AE000519) and conducted in accordance with the National Health and Medical Research Council guidelines (Australia) and ARRIVE guidelines.

### Cell preparation

Human placental tissues were processed, and single-cell suspension was prepared to isolate fetal ECFC and MSC using our previously published protocols.^11-13^ Human term placenta was obtained with written informed consent from healthy women undergoing caesarean deliveries at term (38-39 weeks of gestation) at the Royal Brisbane and Women’s Hospital, as approved by both The University of Queensland and the Royal Brisbane and Women’s Hospital human research ethics committees (RBWH HREC/09/QRBW/14 & UQ 2009000508). The MSCs were derived using villous tissue taken from the term placenta, with care taken to dissect away and remove any contaminating tissue. Cells were cultured and characterized for correct phenotype as previously described.^11^ For *in vivo* stem cells treatment on day of birth, a single dose of 1 × 10^6^ ECFC and 1 × 10^6^ MSC were injected intravenously into the cECFC group and the MSC group were administered with 1 × 10^6^ MSC only.

### Immunohistochemistry

Immunohistochemistry, analysis, and imaging acquisition were conducted as previously reported.^3^^,^^11^^,^^14^ Brain slices containing parietal cortex from the right hemisphere were embedded in paraffin and sectioned at 6 μm. Sections were dewaxed and rehydrated using standard protocols followed by heat-induced epitope retrieval at 90 °C for 20 minutes. Sections were blocked with 5% donkey serum in PBS with 0.5% Triton-X 100 for 1 hour. Primary antibodies (Table S2) were incubated overnight at 4 °C. Slides were washed followed by incubation with species-specific secondary fluorophores for 1 h. Sections were washed, counterstained with 4′,6-diamidino-2 phenylindole, and mounted with Prolong Gold antifade (Molecular Probes, Invitrogen, Australia). Negative control sections without primary antibodies were processed in parallel and immunolabeling was conducted at the minimum in duplicates for all animals.

### Image acquisition and analysis

Images were acquired using a Zeiss Axio Microscope with an Axiocam503 camera and ZEN 2012 software, using either an EC Plan-Neofluar 20x/0.50 M27 (FWD = 2.0 mm) or EC Plan-Neofluar 40×/0.75 M27 (FWD = 0.71 mm) objective. Four images were captured for analysis of each region of interest per section. All imaging and analyses were conducted under blind conditions as per previous studies.^4^^,^^11^^,^^14^ Microglia were manually counted and categorized with respect to morphology.^3^ Density analysis, co-localization, and vessel coverage analyses were undertaken using the threshold function with moments plugin in FIJI (ImageJ; National Institutes of Health, Bethesda, USA).

### Quantitative polymerase chain reaction (qRT-PCR)

Total RNA from frozen parietal cortex samples was isolated using RNeasy Tissue Mini Kit (Qiagen). Yield and quality were determined using a NanoDrop spectrophotometer (ND-1000 system). The expression of the genes of interest was measured using the QuantiNova SYBR Green RT-PCR Kit (Qiagen) on a Qiagen Rotor-Gene Q real-time cycler as per recommendations. The amplified transcripts were quantified with the comparative CT method using GAPDH and β-actin as the normalizing constitutive genes.

### Statistical analysis

Statistical analysis was conducted with GraphPad Prism (GraphPad Software, San Diego, USA). As appropriate, data are presented in figures as mean and SEM. One- or two-way ANOVA with the Tukey post–hoc analyses were performed, and statistical significance was accepted at *P* < .05.

## Results and discussion

### Moderate improvement in gray matter with ongoing perturbations to white matter following cECFC treatment

**Figure 1.**
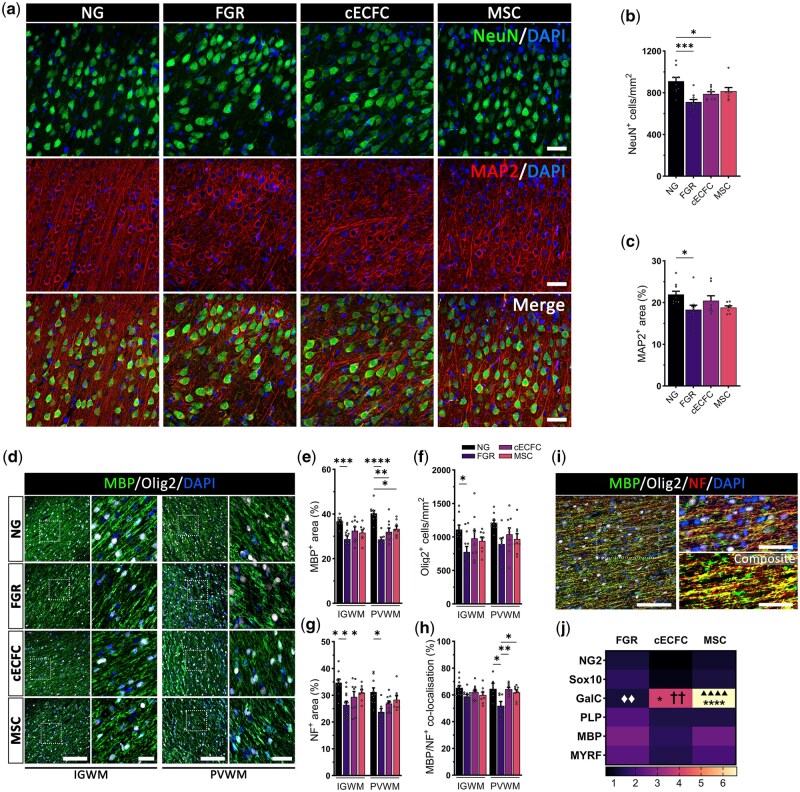
Moderate improvement in neuronal and white matter cell populations at postnatal day 10 following cECFC administration. (A) Untreated fetal growth restriction (FGR) displayed regions void of NeuN^+^ cells and less robust MAP2 labeling in the cortex. cECFC treatment subtly improved NeuN cell counts and displayed normalized MAP2 labeling (Scale bars: 50 µm). (B) Untreated FGR displayed reduced NeuN^+^ cell counts and (C) MAP2^+^ labeled area compared with normally grown (NG). cECFC treated animals displayed a moderate recovery in NeuN^+^ cell count and comparable MAP2^+^ area to NG. (D) myelination (MBP) labeling was less consistent in FGR groups and with more overt hypomyelination in the periventricular white matter (PVWM) (scale bars: low magnification 500 µm, high magnification 100 µm). (E) cECFC showed recovery in the IGWM but reduced MBP was observed in deeper PVWM. (F) Oligodendrocyte (Olig2) was reduced in the IGWM corresponding with a decrease in MBP coverage of NF. (G) Untreated FGR displayed reduced neurofilament (NF) expression in both the intragyral white matter (IGWM) and PVWM. (H) decreased myelination was observed in the untreated FGR relative to all other groups in the PVWM. (I) Co-localization of MBP to NF was assessed using thresholding analysis. Composite image demonstrates regions with hypomyelination (red) (scale bars: low magnification 100 µm, high magnification 50 µm). (J) Examination of myelin regulatory genes found significant upregulation of GalC in cECFC (*c.f. NG, ^♦^*c.f*. untreated FGR, ^†^  *c.f*. MSC) and MSC (*c.f. NG, ^▲^*c.f*. untreated FGR). All values are expressed as mean ± SEM (minimum *n* = 6 for all groups). Two-way ANOVA with Tukey post–hoc test (*P < .05).

Here, we report a decrease in post–mitotic neurons in the parietal cortex (PCtx) of untreated FGR compared with NG (*P* = .0001; Figure 1A and B). Administration of cECFC resulted in a moderate recovery of NeuN^+^-cells but remained significantly lower than NG (*P* = .0361; Figure 1B). Examination of the neuronal cytoskeletal marker MAP2 found reduced levels in untreated FGR when compared with NG (*P* = .0266; Figure 1C). Overall, cECFC treatment displayed mild recovery in neuronal integrity at P10 compared with observations previously reported at P4.^3^ This finding may indicate ongoing neuronal stressors in the FGR brain, with the positive benefits of cECFC treatment diminishing after 4 days post–treatment.

White matter is significantly impacted in FGR,^1^ with studies demonstrating alterations to myelination.^15^ Mild normalization in myelination (MBP), oligodendrocytes (Olig2), and axonal elements (neurofilament; NF) were observed in both the cECFC and MSC-treated FGR in the intragyral white matter (IGWM) (Figure 1D). Examination of the periventricular white matter (PVWM) did not display the same positive effects for MBP labeling, suggestive of ongoing impairment of ventricular white matter structures in untreated (*P* < .0001), cECFC (*P* = .0011), and MSC (*P* = .0145) groups. While the most overt changes in MBP were observed in the PVWM (Figure 1E), no change in Olig2^+^-cell count was found (Figure 1F). Verney et al. reported no difference in Olig2^+^-cells in the presence of non–cystic PVWM injury.^16^ These data indicate that loss in oligodendrocytes alone may not be the significant pathogenic contributor to impaired myelination, rather suggesting an issue with oligodendroglia maturation.^17^ Further studies are required to differentiate lineage progression of oligodendrocytes following treatment. Reduced labeling of neurofilaments (NF) was observed in the white matter of untreated FGR compared with NG (IGWM: *P* = .0010, PVWM: *P* = .0213; Figure 1G). Altered co-localization of MBP with NF proteins was only observed in the PVWM of untreated FGR (*c.f.* NG: *P* = .0142, *c.f.* cECFC: *P* = .0030, *c.f.* MSC *P* = .0405; Figure 1H and I), indicating a region-specific loss in myelin ensheathment of axons which in turn could impact connectivity. These findings contrast with those observed at P4 which demonstrated robust recovery in myelination and recovered pan-oligodendrocyte numbers across all white matter regions examined following cECFC administration.^11^ Expression of key myelin regulating genes found significant upregulation of GalC in cECFC compared with all other groups (*c.f.* NG: *P* = .0274, *c.f.* untreated *P* = .0049, *c.f.* MSC *P* < .0001), and in the MSC group relative to NG (*P* < .001; Figure 1J). These findings indicate the MSC component of our cell therapy may promote formation of myelin sheaths around nerve fibers, but a single dose may not be sufficient to reduce the impacts of FGR. Signaling cues from other cells such as astrocytes are also known to promote adhesion of oligodendrocyte processes to axons and initiate myelination, thus disruption to glial function may also be a key driver to consider with respect to this loss in myelination capacity.^18^

### Cell therapy did not reduce glial activation in PVWM structures

**Figure 2.**
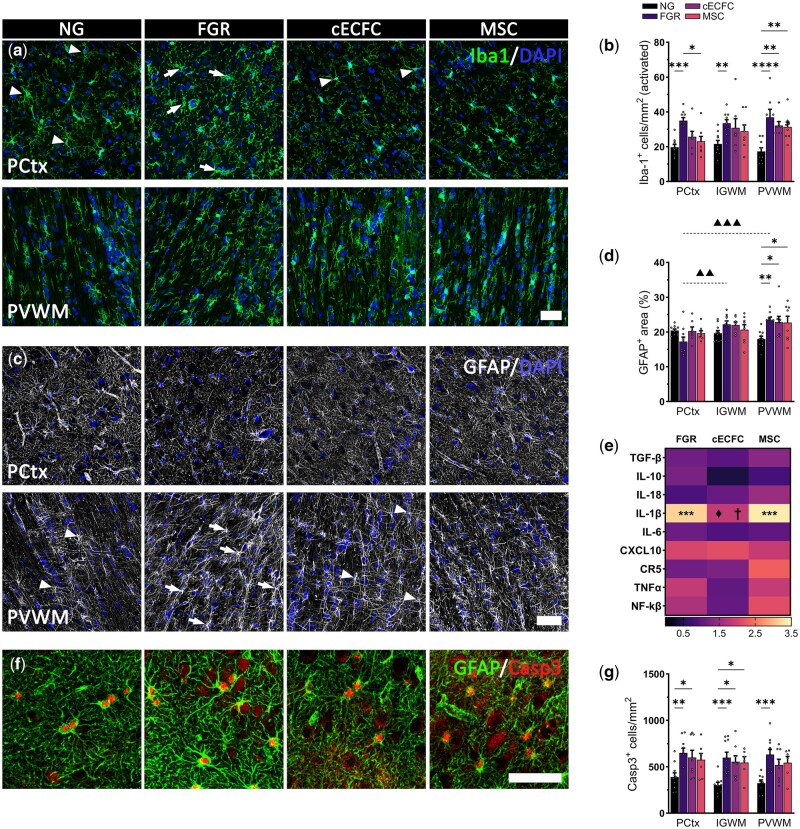
cECFC administration demonstrated regional variation in glial modulation at postnatal day 10. (A) Activated microglial morphology were observed in the parietal cortex (PCtx) and white matter of all fetal growth restriction (FGR) groups (arrows). (B) Quantification of Iba-1^+^ cells with activated morphology showed more evident changes in the periventricular white matter (PVWM) for all FGR groups. (C) Untreated FGR displayed less robust GFAP^+^ labeling in the cortex compared with normally grown (NG) (arrows). Arrowheads indicate normalized astrocytic morphology with long even process extensions. (D) Regional differences were reported for GFAP^+^ labeled area for untreated FGR, with higher astrocyte coverage in both the intragyral white matter (IGWM; ^▲▲^*P* < .01) and PVWM (^▲▲▲^*P* < .001) compared with the cortex. In the PVWM all FGR groups presented increased GFAP^+^ area compared with NG. (E) Heatmap displaying gene expression of previously identified dysregulated inflammatory mediators. Expression levels are relative to NG group. IL-1β was significantly elevated in both the untreated FGR and MSC groups (*P* < .001). cECFC treatment significantly decreased IL-1β expression compared to untreated (^♦^*P* < .05) and MSC (^†^*P* < .05) groups. (F and G) Co-labeling of GFAP and Casp3 showed significantly increased numbers of astrocytes undergoing apoptosis in FGR cohorts. All values are expressed as mean ± SEM (minimum *n* = 6 for all groups). Two-way *ANOVA* with Tukey post–hoc test (*P < .05) (Scale bars: 50 µm).

Microglial activation was normalized in cECFC treated FGR cortex comparable to NG at P10. This effect was diminished when examining white matter, with the PVWM displaying evident increases in Iba-1 positive cells in FGR (*P* = .0006), cECFC (*P* = .0057), and MSC (*P* = .0035) (Figure 2A and B). Assessment of astrocyte populations found less variation in GFAP-positive labeling in the cortex and IGWM across all groups. Region specific increases in GFAP-positive labeling observed in both the IGWM (*P* = .003) and PVWM (*P* = .0002) compared with the cortex for untreated FGR (Figure 2C and D). In the PVWM, we report an evident increase in GFAP labeling associated with reactive morphology in the untreated FGR brain (*P* = .002; Figure 2C and D). While administration of cECFCs or MSCs did not result in a significant reduction in GFAP-positive labeled area, we did observe normalized astrocyte morphology comparable to control brains (Figure 2C and D). A recent study has shown the administration of bone marrow derived-MSCs resulted in increased GFAP in the PVWM of lymph mice at P18, which authors proposed to be neuroprotective.^19^ These findings suggest ventricular white matter regions of FGR brains demonstrate ongoing glial activation at P10, but whether this is neuroprotective or deleterious is to be determined.

We have previously reported a strong association between glial activation and elevated pro-inflammatory mediators in the developing FGR brain.^3^^,^^11^^,^^14^ Examination of classical inflammatory genes demonstrated variable modulatory capacity of our cell therapies. Of note, both the untreated and MSC only groups showed significantly increased IL-1β relative to NG (*P* = .0005 and *P* = .0003 respectively), while cECFC treatment was comparable to controls (Figure 2E). Administration of MSC and cECFCs elevated expression of anti–inflammatory mediators at postnatal day 4,^11^ a finding not observed here at postnatal day 10. Increased apoptosis was observed in untreated FGR compared with NG for all regions examined (PCtx: *P* = .0037, IGWM: *P* = .0009, PVWM: *P* = .0008; Figure 2F and G). Both the cECFC and MSC groups showed lower apoptosis in the PVWM toward levels comparable with NG (Figure 2G). In the cortex, neuronal and astrocytic cells accounted for over 90% of apoptotic cells in all groups, with untreated FGR presenting approximately 45% neuronal compared with 23% in NG (*P* = .0473). The ongoing glial activation, mild modulation of inflammatory mediators and elevated apoptosis may suggest a diminished efficacy of both cECFCs and MSCs therapies by postnatal day 10. We propose an additional dose of cECFCs may be required at postnatal Day 4-5 to modulate the inflammatory environment. Recent studies have shown that multiple doses of MSCs are more effective at modulating inflammation in models of sepsis and cerebral ischemia.^20^^,^^21^

### cECFC treatment improved cerebrovascular structure

**Figure 3.**
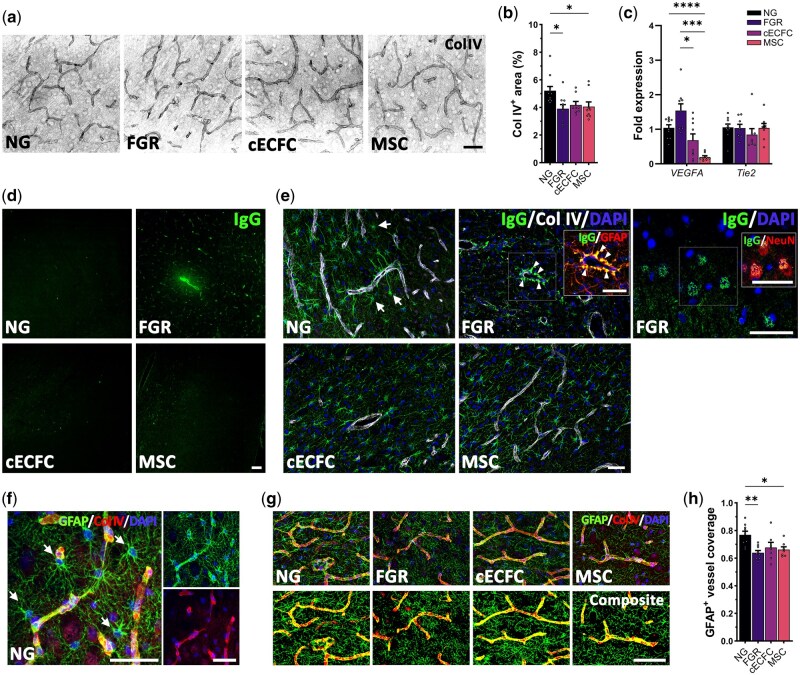
Normalized cerebrovasculature observed in cECFC treated pigs at postnatal day 10. (A) Collagen IV labeling demonstrated truncated vascular structures in untreated fetal growth restriction (FGR). (B) Reduced Col IV labeling in untreated and mesenchymal stromal cell (MSC) only groups compared with normally grown (NG). (C) Gene expression of vascular endothelial protein A (VEGFA) was significantly lowered in both cECFC and MSC compared to untreated FGR. (D) Rare instances of IgG extravasation were observed in FGR groups. (E) Juxtavascular astrocytic uptake of IgG was observed in all groups (arrows), however in untreated FGR the IgG appeared as aggregates at the vascular interface (arrow heads). Neuronal uptake was also observed in untreated FGR (insert). (F) Example of typical juxtavascular astrocyte morphology (arrows) with strong and coherent astrocyte-endfeet vessel ensheathment. (G) Co-localization analysis demonstrated a high degree of astrocyte end-feet interaction with vasculature in all groups, however untreated FGR displayed a slight reduction (H). All values are expressed as mean ± SEM (minimum *n* = 6 for all groups). One-way *ANOVA* with Tukey post–hoc test (*P < .05) (scale bars: 50 µm).

Assessment of cerebrovasculature found no regional differences within phenotypes, we therefore present data as a combination of cortex and white matter. We observed a decrease in vasculature as assessed by basement membrane labeling (Col IV) in FGR compared with NG (*P* = .0159; Figure 3A and B) as previously reported.^4^^,^^11^ The MSC group was also significantly lower than NG (*P* = .048), with no difference reported in the cECFC-treated group (Figure 3B). This finding is supported by recent studies showing that combining MSCs and ECFCs was more effective at enhancing vascularization in ischemic cardiac injury than single-cell approaches, through synergism of MSCs paracrine effects and the angiogenic effects of ECFCs.^22-24^ Examination of vascular endothelial protein A (VEGFA) gene expression found significantly increased levels in untreated FGR relative to cECFC (*P* = .0344) and MSC (*P* = .0005). Prior study showed elevated VEGF in cord blood of birth asphyxiated newborns was significantly elevated in those that later developed encephalopathy,^25^ thus elevated levels in the brain may be associated with ongoing injury. Of note, the MSC only group showed significantly downregulated VEGFA compared with NG (*P* < .0001; Figure 3C). Previous reports indicate MSCs can down-regulate VEGF and contribute to the inhibition of ­angiogenesis,^26^ which may explain the maintained lower levels of ColIV in the MSC only group. No significant change in Tie2 gene expression suggests vascular quiescence across all FGR groups relative to NG (Figure 3C).

The neurovascular unit (NVU) is critical in regulating the brain microenvironment. We and others have previously reported that altered cell interaction at the NVU contributes to loss in BBB integrity in FGR pig and sheep.^4^,^6^ We therefore examined the BBB at P10 in FGR brains. Qualitatively, we observed limited extravasation in both untreated and treated FGR groups, with serum proteins predominantly restricted to the lumen or taken up by perivascular astrocytes (Figure 3D and E), indicating recovered NVU integrity across all FGR groups compared with observations at postnatal day 4. Of note, untreated FGR demonstrated aggregated uptake of IgG in astrocytic-endfeet at the vessel interface, which may indicate a deficit in astrocytic sequestering of serum proteins when compared with NG and treated FGR (Figure 3E). This additional stressor could also impact astrocyte-neuron support and contribute to the ongoing perturbations observed in neuronal and white matter structures. In the healthy brain, astrocytes demonstrate consistent endfeet contact with the vasculature (Figure 3F). Here, we found decreased astrocytic-endfeet interaction with vasculature in the untreated FGR (*P* = .0033) and MSC (*P* = .0288) groups (Figure 3G and H). Bell et al. report increased astrocyte-vessel contact following ECFC treatment in the FGR sheep, though this study used a higher dosage (10^7^ cells) of a single cell type derived from cultured human UCB.^5^ These findings suggest a single dose of cECFC or MSCs may have limited capacity for long-term modulation of the NVU.

## Conclusion

In summary, we observed moderate neuroprotective capacity of cECFC administration in FGR brain at postnatal day 10 when compared with acute outcomes reported at postnatal day 4. PVWM regions displayed persistent cellular and structural pathology, possibly associated with ongoing glial activation and limited modulation of the inflammatory environment. This decrease in efficacy is likely associated with a decline in the number of administered cells remaining in the treated brain due to clearance^27^^,^^28^ in addition to premature senescence,^29^ and diminished trophic and immunomodulatory potential for cells to afford ongoing neuroprotection. As these positive modulatory effects decrease, the adverse brain environment may recapitulate detrimental processes such as the re-establishment of a pro-inflammatory environment. Further studies must be undertaken to examine the benefits of multiple doses and concentrations to improve efficacy and allow for sustainable long-term outcomes. Critically, our study highlights the need for longer-term assessment of reparative potential following cell therapy in pre–clinical animal models of neonate brain injury.

## Supplementary Material

szag019_Supplementary_Data

## Data Availability

The data generated in this study are available within the article and its supplementary data files.
