# Supplementary material for: Moderate neuroprotection of combination cell therapy in fetal growth restricted newborns at postnatal day 10
Source: Stem Cells Transl Med. 2026 Apr 20;15(5):szag019. doi: 10.1093/stcltm/szag019 (PMC13120858; doi:10.1093/stcltm/szag019)
Supplement: szag019_Supplementary_Data [file szag019_supplementary_data.zip › Supplementary information_all.docx]

**Moderate neuroprotection of combination cell therapy in fetal growth restricted newborns at postnatal day ten**

Running head: cECFC treatment affords moderate neuroprotection in FGR

**Supplementary information**

Kirat K Chand^1^, Kate Beecher^1^, Rachel Nano^2^, Seen-Ling Sim^2^, Jane Sun^2^, Peytn Stokes-Marshall^1^, Lillian Macfarlane^1^, John Luff^1^, Hannah Musco^1^, Paul B Colditz^1,4^, Kiarash Khosrotehrani^2^, Jatin Patel^2,3^, Julie A Wixey^1^

^1^UQ Centre for Clinical Research, Faculty of Medicine, The University of Queensland, Brisbane, QLD, Australia.

^2^Frazer Institute, The University of Queensland, Woolloongabba, QLD, Australia.

^3^Faculty of Health, Queensland University of Technology, School of Biomedical Sciences, Brisbane, QLD, Australia.

^4^Perinatal Research Centre, Royal Brisbane and Women’s Hospital, Brisbane, QLD, Australia.

**Author Contributions:** All authors reviewed and approved the final manuscript.

Author 1: K.K.C: Conceptualization, methodology, formal analysis, investigation, data curation, writing- original draft and reviewing and editing.

Author 2: K.B.M: Methodology, formal analysis, data curation.

Author 3: R.N: Methodology.

Author 4: S-L.S: Methodology.

Author 5: J.S: Methodology.

Author 6: P.S-M: Methodology, formal analysis.

Author 7: L.M.: Methodology, formal analysis.

Author 8: J.L.: Methodology

Author 9: H.M: Methodology

Author 10: P.B.C: Funding acquisition, writing- reviewing and editing.

Author 11: K.K: Funding acquisition, methodology, resources, writing- reviewing and editing.

Author 12: J.P: Conceptualization, funding acquisition, methodology, resources, writing- reviewing and editing.

Author 13: J.A.W. Conceptualization, funding acquisition, methodology, project administration, writing- reviewing and editing.

**Acknowledgements:** We would like to thank the women at the Royal Brisbane and Women’s Hospital for kindly donating their placentas for this study and to Ruben McQuarrie for his volunteer work and assistance with data analysis. We would also like to thank the Flow Cytometry Facility at the Translational Research Institute for their kind assistance.

**Funding:** J.P. salary was supported by the Future Leader Fellowship funded by the National Heart Foundation of Australia. K.K. salary was supported by the NHMRC Career Development Fellowship (APP1125290) funded by the Australian Government. J.A.W salary was supported by an NHMRC grant (2017225). A Cerebral Palsy Alliance grant (PRG05119) and Royal Brisbane and Women’s Hospital Foundation grant supported this work. Funding bodies did not influence the design of the study nor collection, analysis, interpretation of data, and drafting of the manuscript.

**Keywords:** Fetal Growth Restriction, Stem Cells, Brain injury, Neonate

**Conflict of interest:** JP and KK are lead inventors on patents to isolate human ECFC and MSC from the placenta. This has now been licence to HAON Life Sciences.

**Data availability:** The data generated in this study are available within the article and its supplementary data files.

| **Supplementary Table 1:** Physiological parameters in FGR and NG piglets | | | | |  |  |
| --- | --- | --- | --- | --- | --- | --- |
|  | **NG (n=10)** | **FGR (n=10)** | **cECFC (n=8)** | **MSC (n=8)** | | |
| Body weight (kg) – P1 | 1.65 ± 0.05 | 0.86 ± 0.04**** | 0.89 ± 0.05**** | 0.87 ± 0.05**** | | |
| Bodyweight (kg) – P10 | 2.66 ± 0.15 | 1.36 ± 0.09**** | 1.41 ± 0.12**** | 1.21 ± 0.15**** | | |
| Brain: body weight (g/kg) | 14.53 ± 0.54 | 24.68 ± 1.52*** | 24.04 ± 1.68*** | 28.37 ± 2.68**** | | |
| Liver: body weight (g/kg) | 28.30 ± 0.91 | 31.15 ± 1.23 | 30.63 ± 1.89 | 30.65 ± 1.82 | | |
| Brain: Liver (g/g) | 0.52 ± 0.03 | 0.81 ± 0.07* | 0.81 ± 0.08* | 0.94 ± 0.10*** | | |
| Heart: body weight (g/kg) | 7.30 ± 0.31 | 7.72 ± 0.25 | 7.20 ± 0.40 | 7.02 ± 0.36 | | |
| Lung: body weight (g/kg) | 15.22 ± 0.43 | 14.17 ± 0.69 | 13.64 ± 0.65 | 14.66 ± 1.16 | | |
| Kidney: body weight (g/kg) | 3.81 ± 0.20 | 3.62 ± 0.14 | 4.30 ± 0.10 | 4.22 ± 0.21 | | |
| Mean Temperature (^o^C) | 39.05 ± 0.13 | 39.07 ± 0.11 | 38.96 ± 0.19 | 38.93 ± 0.23 | | |
| All values presented as mean ± SEM *p < 0.05, **p < 0.01, ***p < 0.001, ****p < 0.0001 | | | | | |  |

**Supplementary Table 2:** Antibodies utilised in study

| **Primary Antibody** | **Host** | **Dilution** | **Catalogue#** |
| --- | --- | --- | --- |
| Anti-human CD34-PE | Mouse | 1:25 | Bio-Rad (MCA1578PE) |
| Anti-human CD45-FITC | Mouse | 1:25 | BioLegend (304006) |
| Anti-human CD31-V450 | Mouse | 1:30 | BD Biosciences (561653) |
| 7AAD |  | 1:40 | BD Pharmingen (559925) |
| Microtubule-associated protein 2 (MAP2) | Mouse | 1:500 | Sigma-Aldrich (M4403) |
| Cleaved Caspase-3 | Rabbit | 1:500 | Cell Signaling (#9661) |
| Collagen IV (Col IV) | Rabbit | 1:1000 | Abcam (ab6586) |
| Glial fibrillary acidic protein (GFAP) | Mouse | 1:1000 | Sigma (G3893) |
| Glial fibrillary acidic protein (GFAP) | Rabbit | 1:2000 | DAKO (Z0334) |
| IgG | Goat | 1:1000 | JIR (114-005-003) |
| Ionised calcium binding adaptor molecule-1 (Iba-1) | Goat | 1:1000 | Abcam (ab5076) |
| Microtubule-associated protein 2 (MAP2) | Mouse | 1:500 | Sigma-Aldrich (M4403) |
| Myelin Binding Protein (MBP) | Rat | 1:1000 | Abcam (7349) |
| Neurofilament (NF) | Mouse | 1:500 | Abcam (ab134306) |
| Neuronal Nuclei (NeuN) | Rabbit | 1:1000 | Abcam (ab177487) |
| Oligodendrocyte marker 2 (Olig2) | Rabbit | 1:1000 | Genetex (GTX132732) |
| **Secondary Antibody** |  |  |  |
| α-Rat Alexafluor 488 | Donkey | 1:1000 | Invitrogen (A-21208) |
| α-Rabbit Alexafluor 488 | Donkey | 1:1000 | Invitrogen (A-21206) |
| α-Goat Alexafluor 568 | Donkey | 1:1000 | Invitrogen (A-11057) |
| α-Mouse Alexafluor 568 | Donkey | 1:1000 | Invitrogen (A10037) |
| α-Rabbit Alexafluor 594 | Donkey | 1:1000 | Invitrogen (A-21207) |
| α-Mouse Alexafluor 647 | Donkey | 1:1000 | Invitrogen (A-31571) |
